# High hemoglobin glycation index is associated with increased risk of diabetes: A population-based cohort study in China

**DOI:** 10.3389/fendo.2023.1081520

**Published:** 2023-02-27

**Authors:** Lu Lin, Anping Wang, Xiaomeng Jia, Haibin Wang, Yan He, Yiming Mu, Jingtao Dou

**Affiliations:** ^1^ Department of Endocrinology, The First Medical Center, Chinese People's Liberation Army (PLA) General Hospital, Beijing, China; ^2^ Department of Endocrinology, Hainan General Hospital, Hainan Affiliated Hospital of Hainan Medical University, Haikou, China; ^3^ Center for Endocrine Metabolism and Immune Disease, Beijing Luhe Hospital, Capital Medical University, Beijing, China; ^4^ Department of endocrinology, First Affiliated Hospital of Zhengzhou University, Zhengzhou, Henan, China; ^5^ Department of Epidemiology and Biostatistics, School of Public Health, Capital Medical University, Beijing, China; ^6^ Municipal Key Laboratory of Clinical Epidemiology, Beijing, China

**Keywords:** biological variation, diabetes mellitus, glycated hemoglobin A1c, hemoglobin glycation index, risk factor

## Abstract

**Purpose:**

The hemoglobin glycation index (HGI) quantifies the mismatch between glycated hemoglobin A1c and average glycemia among individuals. Currently, it is unknown the potential role of HGI in exhaustively evaluating the progression of glucose metabolism/the risk of developing diabetes mellitus. Therefore, this study aimed to investigate the association between HGI and the risk of incident diabetes.

**Methods:**

A total of 7,345 participants aged at least 40 years and without diabetes were divided into three groups according to the tertile of their baseline HGI level and followed for a median of 3.24 years to track new-onset diabetes. Using multivariate Cox regression analyses, we explored the association between the HGI, both categorized and continuous, and incident diabetes.

**Results:**

During follow-up, 742 subjects (263 males and 479 females) developed diabetes mellitus. Higher HGI was associated with an increased risk of diabetes, even when adjusted for confounding factors, and every standard deviation increase in HGI was associated with a significant risk increase of 30.6% for diabetes (hazard ratio 1.306, 95% confidence interval 1.232–1.384).

**Conclusions:**

Participants with a higher HGI were at a higher risk of future diabetes, irrespective of their glycemic conditions. Consequently, HGI may be employed to identify individuals at high risk for diabetes.

## Introduction

During the past two decades in clinical practice, glycated hemoglobin (HbA1c) has been universally applied for screening, diagnosis, and glycemic control monitoring in patients with diabetes (1, 2). HbA1c is considered an important biomarker indicating average blood glucose levels over a period of 8–12 weeks (3). However, HbA1c is not a one-size-fits-all indicator for assessing chronic glycemia, which ignores inter-individual variations in the relationship between HbA1c and average glucose (4, 5). In 2002, Hempe et al. developed and validated the hemoglobin glycation index (HGI) to quantify the inter-individual consistent disparity between HbA1c and the mean blood glucose (MBG) level (6).

The HGI is a metric describing inter-individual biological variation of HbA1c or individual propensity for glycation of hemoglobin, which is another major factor affecting HbA1c results besides blood glucose concentration (3, 7). The HGI is computed as measured HbA1c minus predicted HbA1c. The predicted HbA1c was initially calculated by inserting the date-matched MBG into a linear regression equation derived from the measured HbA1c and MBG. Some studies have confirmed that using fasting plasma glucose (FPG) to assess predicted HbA1c and calculate HGI is feasible (8, 9). Most prior studies have primarily reported the association between HGI and diabetes complications. An elevated HGI might promote diabetes complications through inflammation and the formation of advanced glycation end products (AGEs) (10, 11). Existing literature suggests that the HGI is a strong predictor of the risk of diabetes complications among patients with either type 1 or type 2 diabetes (12, 13).

However, only a few studies have investigated the impact of HGI on glucose metabolism. A recent study in Italy demonstrated that patients without diabetes with a high HGI had higher fasting insulin levels and more severe insulin resistance than those with a low HGI (14). Moreover, a higher HGI appears to be related to older age, obesity, and dyslipidemia, which are risk factors for diabetes (10, 15, 16). Based on previous studies (10, 14–16), we hypothesized that individuals with a high HGI might have an increased risk of developing diabetes. Therefore, the current study aimed to investigate the association between the HGI and the incidence of diabetes among a population in China, using a prospective cohort study design.

## Methods

### Study design and participants

The Risk Evaluation of Cancers in Chinese Diabetic Individuals: A longitudinal (REACTION) study was a multicenter population-based prospective cohort study investigating the association between abnormal glucose metabolism and increased risk of cancer among the population of China. The experimental design of the REACTION study has been published elsewhere (17, 18).

**Figure 1:**
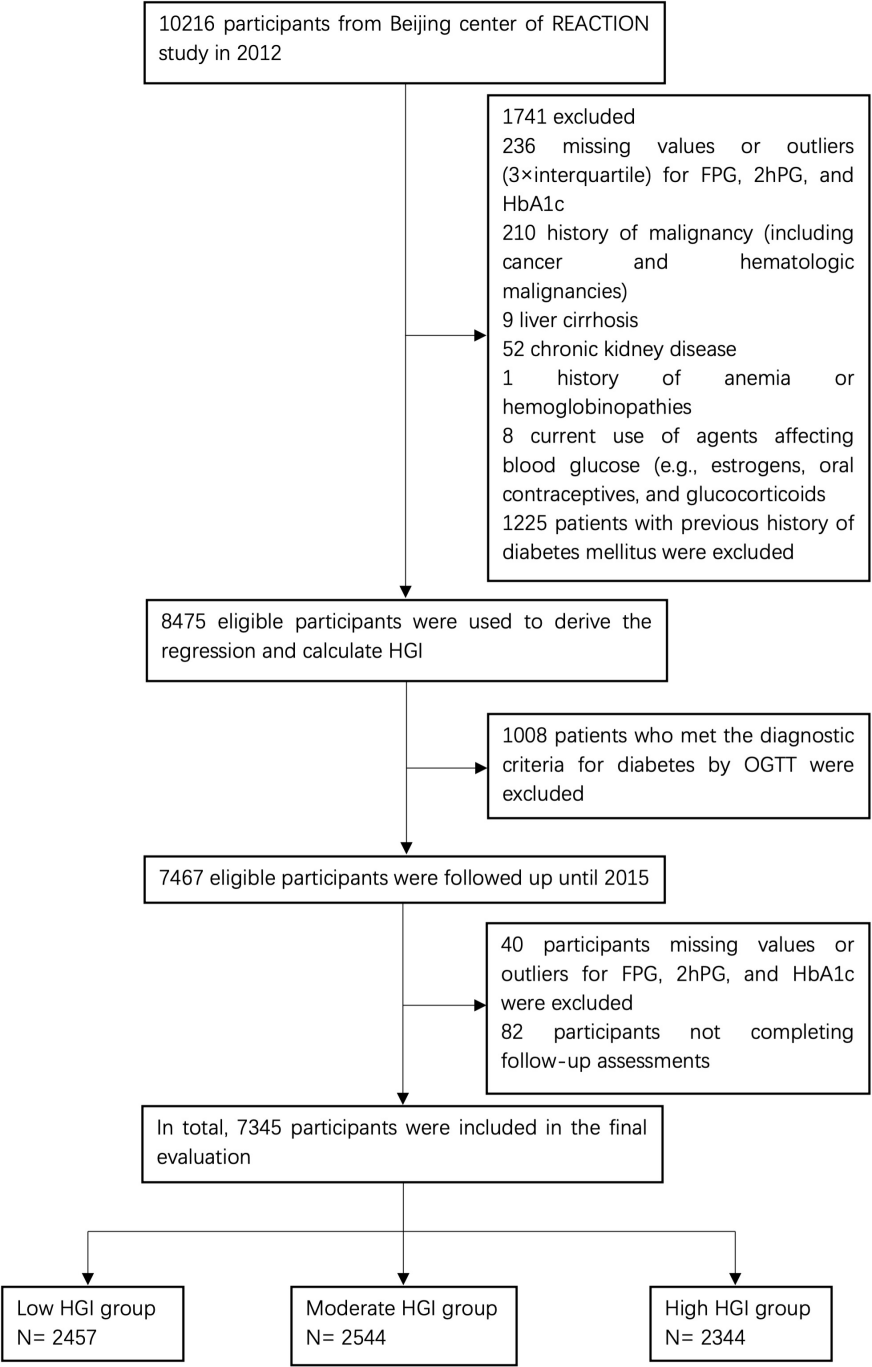
Study flowchart of participants. FPG, fasting plasma glucose; 2hPG, 2-h plasma glucose; HbA1c, hemoglobin A1c; HGI, hemoglobin glycation index; OGTT, oral glucose tolerance test.

This present study was retrospective, and analysis was performed based on the data from a subcenter (the Pingguoyuan community of Beijing) of the REACTION study, which was conducted by the Department of Endocrinology of First Medical Center of Chinese PLA General Hospital. From January to August 2012, 10,126 individuals aged ≥40 years were recruited from the study location, of whom 2,749 participants did not meet the inclusion criteria and were therefore excluded at the baseline visit. In total, 7,467 eligible participants without diabetes were consecutively enrolled and followed up from April to October 2015. Of the 7,467 participants, 82 (1.1%) were lost to follow-up; thus, the study achieved a response rate of 98.6%. In addition, 40 patients with missing data on FPG, 2-h plasma glucose (2hPG), or HbA1c at follow-up were excluded. Consequently, 7,345 participants were included in the final evaluation (**Figure 1**). This REACTION study program was approved by the Medical Ethics Committee of Shanghai Jiaotong University (No. 2011-14). All procedures were performed according to the tenets of the revised (1983) Declaration of Helsinki. All participants provided written informed consent at each study visit.

### Data collection and laboratory measurements

Following the standardized study protocol, data were collected *via* detailed questionnaires, physical examination, and blood samples during baseline and follow-up visits at a local clinic. Trained clinical staff administered standardized questionnaires and conducted in-person interviews to collect information on demographic data and medical, family, and medication history. Blood pressure, height, body weight, and waist circumference were measured according to standard procedures, and body mass index (BMI) was calculated as weight (kg)/height squared (m^2^).

At each clinic visit, a 75-g oral glucose tolerance test (OGTT) was performed after collecting a fasting blood sample. FPG, HbA1c, fasting lipids, and 2hPG levels were also measured. After collecting venous blood, all samples were immediately placed on ice to maintain stability. Thereafter, the samples were instantly transported to the laboratory at the First Medical Centre of Chinese PLA General Hospital and processed within 2 h of blood collection. For plasma glucose (including FPG and 2hPG), blood samples were collected in tubes containing sodium fluoride and measured using the hexokinase method. HbA1c was measured using high-performance liquid chromatography (VARIANT II system, Bio-Rad, Hercules, CA). Total cholesterol (TC), triglycerides (TG), high-density lipoprotein cholesterol (HDL-C), and low-density lipoprotein cholesterol (LDL-C) levels were determined using an auto-analyzer (ARCHITECT c16000 System; Abbott Laboratories, Chicago, IL). The quality control protocol for laboratory assays has been published in detail elsewhere (17).

The diagnosis of dysglycemia (including pre-diabetes or diabetes) was based on OGTT, conforming to the American Diabetes Association criteria (1). Pre-diabetes was defined as follows: FPG: 100–125 mg/dL (5.6–6.9 mmol/L); or 2hPG during 75 g OGTT: 140–199 mg/dL (7.8–11.0 mmol/L). Diabetes was defined as: documented diagnosis of diabetes in medical records or taking glucose-lowering medications; FPG ≥126 mg/dL (7.0 mmol/L); or 2hPG ≥200 mg/dL (11.1 mmol/L) during 75g OGTT. Normoglycemia was described as FPG <100 mg/dl (5.6 mmol/L) with 2hPG <140 mg/dl (7.8 mmol/L) during 75g OGTT. The primary study outcome was the occurrence of diabetes, defined as diagnosed (i.e., physician-diagnosed diabetes or use of antidiabetic medication during follow-up) or undiagnosed (based on the above diabetes criteria).

### Calculation of the HGI

**Figure 2:**
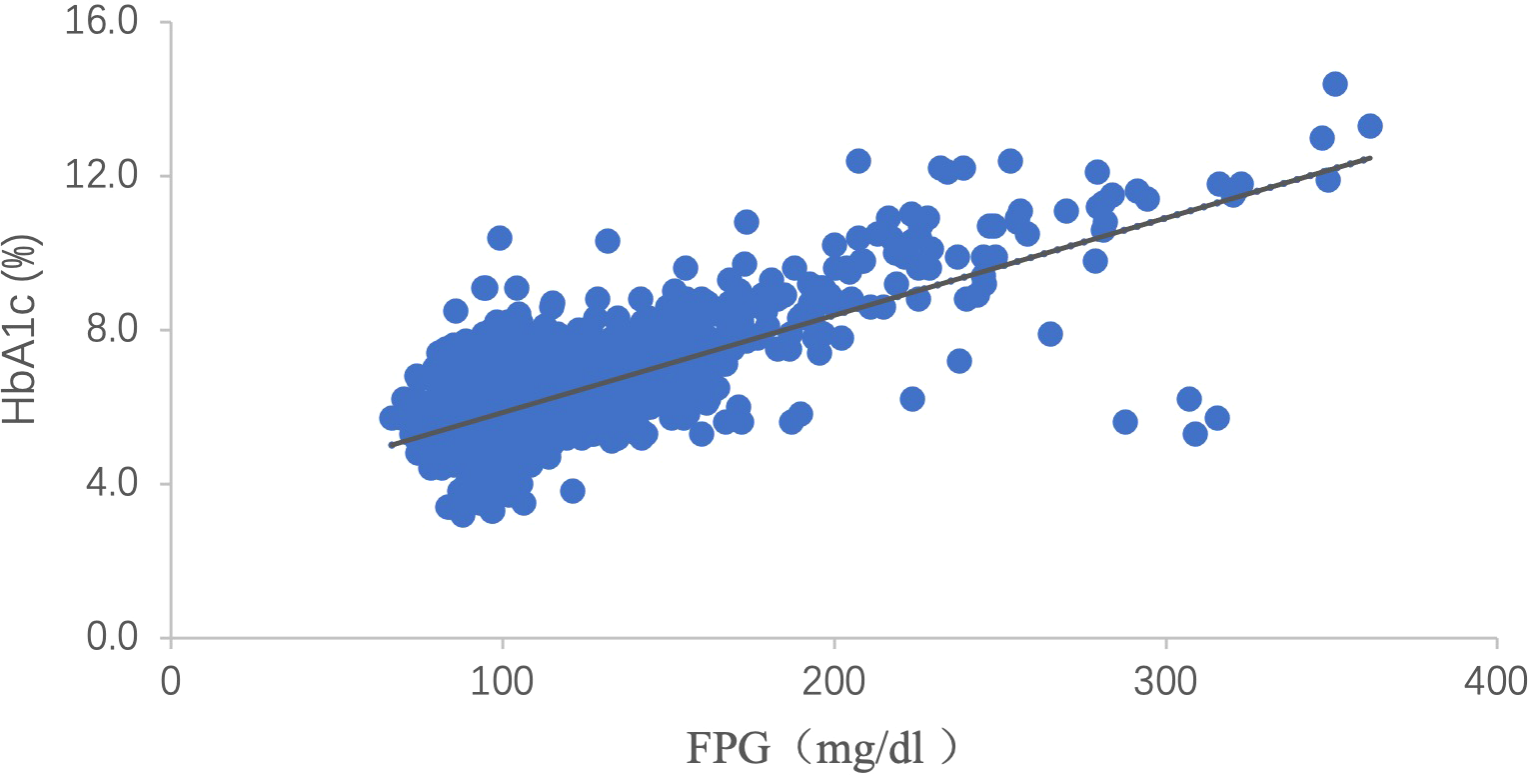
Scattergram of HbA1c versus FPG. There is a clear linear relation between HbA1c and FPG [HbA1c (%) = 3.335 + 0.025 FPG (mg/dL), *P <*0.001, R^2 =^ 0.528]. HbA1c: hemoglobin A1c; FPG: fasting plasma glucose.

A linear regression equation between FPG and HbA1c was established using data from the baseline population of 8,475 participants who met the inclusion criteria (**Figure 2**). The predicted HbA1c level was calculated by imputing FPG into the following equation: HbA1c (%) = 3.335 + 0.025 FPG (mg/dL). Then, the baseline HGI was calculated by subtracting the measured HbA1c from the predicted HbA1c level (8, 9). Tertile cut-off points were determined from the baseline HGI and applied to the study subset of participants without diabetes (n = 7,345). Subsequently, the final study cohort was classified into low (≤ -0.1480), moderate (-0.1480 to 0.1675), and high (>0.1675) HGI groups.

### Statistical analysis

Continuous variables were expressed as mean ± standard deviation (SD) for normally distributed variables or as medians (interquartile range) for non-normally distributed variables. Group comparisons were analyzed using a one-way analysis of variance or the Kruskal–Wallis rank sum test for normally or non-normally distributed data, respectively. Proportions for categorical variables were compared using the chi-squared test or Fisher’s exact test. Subgroup analyses were also performed by baseline glucometabolic status (normoglycemia and pre-diabetes based on OGTT results).

The time to diabetes event was defined as the time from baseline to the reported date of diagnosis obtained at the follow-up visit. For incident diabetes cases diagnosed at the follow-up visit, we inserted the estimated time to diabetes event (using baseline and follow-up values) as the first date at which one of the values met the diagnostic criteria. For instance, if a participant was diagnosed with diabetes using an FPG of 9 mmol/L at a follow-up visit and their FPG at baseline was 5 mmol/L, the incident date was set as half the time of follow-up ([7 – 5]/[9 – 5] = 2/4 = 0.5). The relationship between the incidence of diabetes and the HGI was examined using multivariate Cox proportional hazards regression analyses. The test for trend was performed to ascertain the statistical significance of the trends observed. In all models, covariates were chosen for both their clinical relevance and univariate correlation with the outcome of interest. Owing to the collinearity between the HGI and HbA1c (r = 0.694), none of the models included both the HGI and HbA1c. In addition, the HGI and HbA1c were evaluated per SD change to facilitate a comparison of their strengths in association with the incidence of diabetes. Hazard ratios (HRs) were reported with corresponding 95% confidence intervals (CIs), and statistical significance was set at p<0.05 (two-sided). The Statistical analyses were performed using the Statistical Package for the Social Sciences (version 26.0; IBM, Armonk, NY).

## Results

### Baseline general characteristics of patients in the three HGI groups

The baseline characteristics of the study population according to the HGI category are shown in **Table 1**. Among the 7,345 participants, the mean (SD) baseline age was 56.5 (7.6) years, and 4,885 (66.5%) were female. Participants with high HGI were more likely to be older, have a higher BMI, and have an adverse lipid profile (all *P* for trend <0.001). The same trends were also observed among the normal glycemia and pre-diabetes subgroups (**Tables S1**, **S2**).Even though more participants in the high HGI group had hypertension, diastolic blood pressure (DBP) decreased with HGI tertile (*P* for trend <0.001). There was a stepwise increase in the HbA1c levels in the order of increasing HGI levels owing to the method used to calculate the HGI; however, FPG levels decreased. Compared with low and moderate HGI groups, participants in the high HGI group had significantly higher 2hPG levels in the total study population and pre-diabetes subgroup (**Tables 1**, **S2**).

**Table 1:** Baseline characteristics of 7,345 participants in 2011.

Variables	Total	Low HGI	Moderate HGI	High HGI	Statistical value	*P* value
Participants, *n*	7, 345	2, 457	2, 544	2, 344		
Mean HGI	0.0010 (-0.2370, 0.2402)	-0.3370 (-0.4920, -0.2357)	0.0075 (-0.0680, 0.0839)	0.3597(0.2540, 0.5164)		
Age (years)	56.5 ± 7.6	55.7 ± 7.4	56.6 ± 7.7	57.4 ± 7.6	28.553	<0.001
Female, n (%)	4885(66.5)	1491 (60.7)	1773 (69.7)	1621 (69.2)	56.380	<0.001
BMI (kg/m^2^)	25.5 ± 3.4	25.2 ± 3.3	25.5 ± 3.3	25.8 ± 3.5	19.858	<0.001
Waist circumference (cm)	83.0 ± 8.8	83.1 ± 8.7	82.8 ± 8.7	83.2 ± 9.0	1.120	0.298
SBP (mmHg)	130.3 ± 16.1	130.5 ± 16.2	129.7 ± 16.0	130.8 ± 16.1	3.265	0.038
DBP (mmHg)	75.5 ± 9.5	76.7 ± 9.6	75.2 ± 9.4	74.7 ± 9.5	31.933	<0.001
Family history of diabetes, n (%)	1772 (24.1)	595(24.2)	625 (24.6)	552(23.5)	1.049	0.902
History of hypertension, n (%)	1971 (26.8)	653(26.6)	647(25.4)	671 (28.6)	6.463	0.039
Antihypertensive medication, n (%)	1626 (22.1)	524 (21.3)	541 (21.3)	561 (23.9)	6.445	0.040
Lipid-lowering medication, n (%)	195 (2.7)	36 (1.5)	83 (3.3)	76 (3.2)	20.221	<0.001
TC (mmol/L)	5.23 ± 0.98	5.07 ± 0.93	5.27 ± 1.00	5.35 ± 0.98	51.129	<0.001
TG (mmol/L)	1.28 (0.91, 1.80)	1.22 (0.88, 1.71)	1.27 (0.90, 1.82)	1.33 (0.95, 1.87)	27.497	<0.001
HDL-C (mmol/L)	1.40 (1.18, 1.66)	1.40 (1.18, 1.66)	1.42 (1.20, 1.66)	1.40 (1.18, 1.68)	2.050	0.359
LDL-C (mmol/L)	3.20 ± 0.80	3.08 ± 0.78	3.22 ± 0.79	3.29 ± 0.81	44.794	<0.001
HbA1c (%)	5.8 ± 0.5	5.4 ± 0.3	5.8 ± 0.2	6.2 ± 0.4	2537.421	<0.001
FPG (mg/dL)	97 ± 9	99 ± 10	96 ± 9	95 ± 9	87.036	<0.001
2hPG (mg/dL)	123 ± 29	122 ± 29	122 ± 29	125 ± 31	8.899	<0.001
Pre-diabetes based on OGTT, n (%)	3239 (44.1)	1221 (49.7)	1035 (40.7)	983 (41.9)	46.689	<0.001

*Data are expressed as mean ± standard deviation for normally distributed variables, median (P_25_, P_75_) for non-normally distributed variables, and number (%) for categorical variables.

HGI, hemoglobin glycation index; BMI, body mass index; SBP, systolic blood pressure; DBP, diastolic blood pressure; TC, total cholesterol; TG, triglycerides; HDL-C, high-density lipoprotein cholesterol; LDL-C, low-density lipoprotein cholesterol; HbA1c, glycated hemoglobin A1c; FPG, fasting plasma glucose; 2hPG: 2-h plasma glucose; OGTT, oral glucose tolerance test.

### Association between baseline HGI and incident diabetes

**Figure 3:**
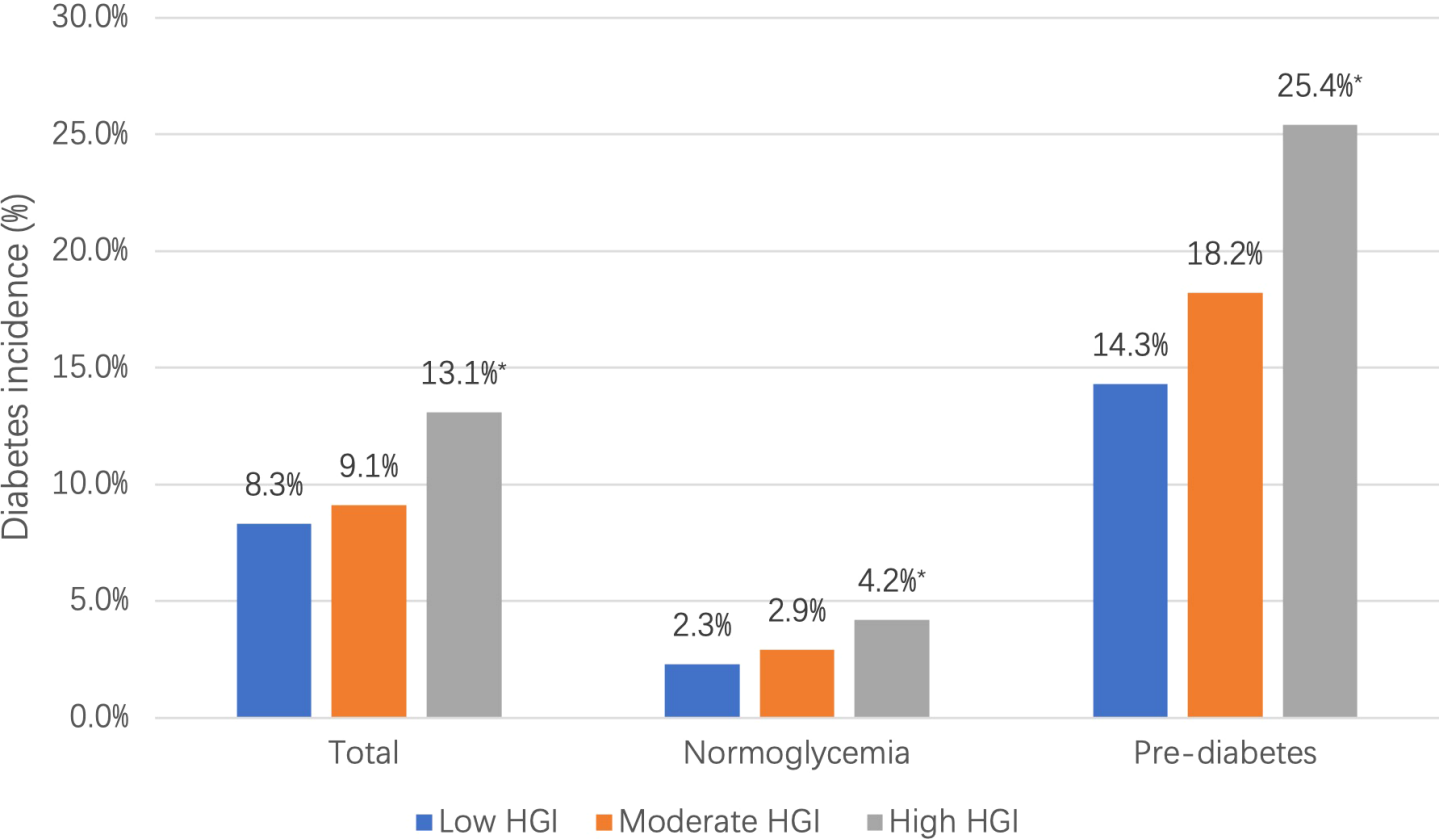
Incidence of diabetes for HGI categories by baseline glucometabolic status ^#^ Glucometabolic status is assessed using oral glucose tolerance test results (including fasting plasma glucose and 2-h plasma glucose). *compared with low HGI group, *P <*0.05 HGI: hemoglobin glycation index.

During the follow-up period (median 3.24 years), 742 participants (10.1%) developed diabetes, with the highest incidence in the high HGI group (13.1%), followed by the moderate (9.1%) and low groups (8.3%). Similarly, the same results were observed in the subgroup analyses by baseline glucometabolic status (**Figure 3**)

To assess for relationships between variables and diabetes incidence, HGI was analyzed both as a categorical and a continuous variable. **Table 2** shows the adjusted HRs for incident diabetes by the HGI group, with the low HGI group as the reference. Multivariate analysis revealed that high HGI was associated with an increased risk of diabetes (models 1 and 2) after adjusting for age, sex, BMI, systolic blood pressure (SBP), DBP, history of hypertension, antihypertensive medication use, TC, TG, and LDL-C. Further adjustment for FPG and 2hPG (model 3) strengthened this association, with adjusted HRs for diabetes reaching 1.366 (95% CI, 1.129-1.653) and 1.903 (95% CI, 1.584–2.288) for the moderate and top tertiles of HGI, respectively, when compared with the bottom tertile (*P* for trend <0.001).

**Table 2:** Adjusted hazard ratios for incident diabetes according to the HGI category at baseline.

Group	Case/overall	Crude model	Model 1 (HR 95% CI)	Model 2 (HR 95% CI)	Model 3 (HR 95% CI)
Low HGI	203/2457	Reference	Reference	Reference	Reference
Moderate HGI	232/2544	1.105 (0.915-1.334)	1.069 (0.885-1.292)	1.048 (0.867-1.267)	1.366 (1.129-1.653)
High HGI	307/2344	1.624 (1.360-1.939)	1.539 (1.286-1.840)	1.468 (1.226-1.757)	1.903 (1.584-2.288)
*P* for trend		<0.001	<0.001	<0.001	<0.001

Model 1 was adjusted for age and sex. Model 2 was adjusted for the variables in model 1 plus body mass index, systolic blood pressure, diastolic blood pressure, hypertension, antihypertensive medication, total cholesterol, triglycerides, and low-density lipoprotein cholesterol. Model 3 was adjusted for all variables in model 2 plus the baseline fasting plasma glucose and 2-h plasma glucose at the OGTT. HGI, hemoglobin glycation index; HR, hazard ratios; CI, confidence interval; OGTT, oral glucose tolerance test.

**Figure 4:**
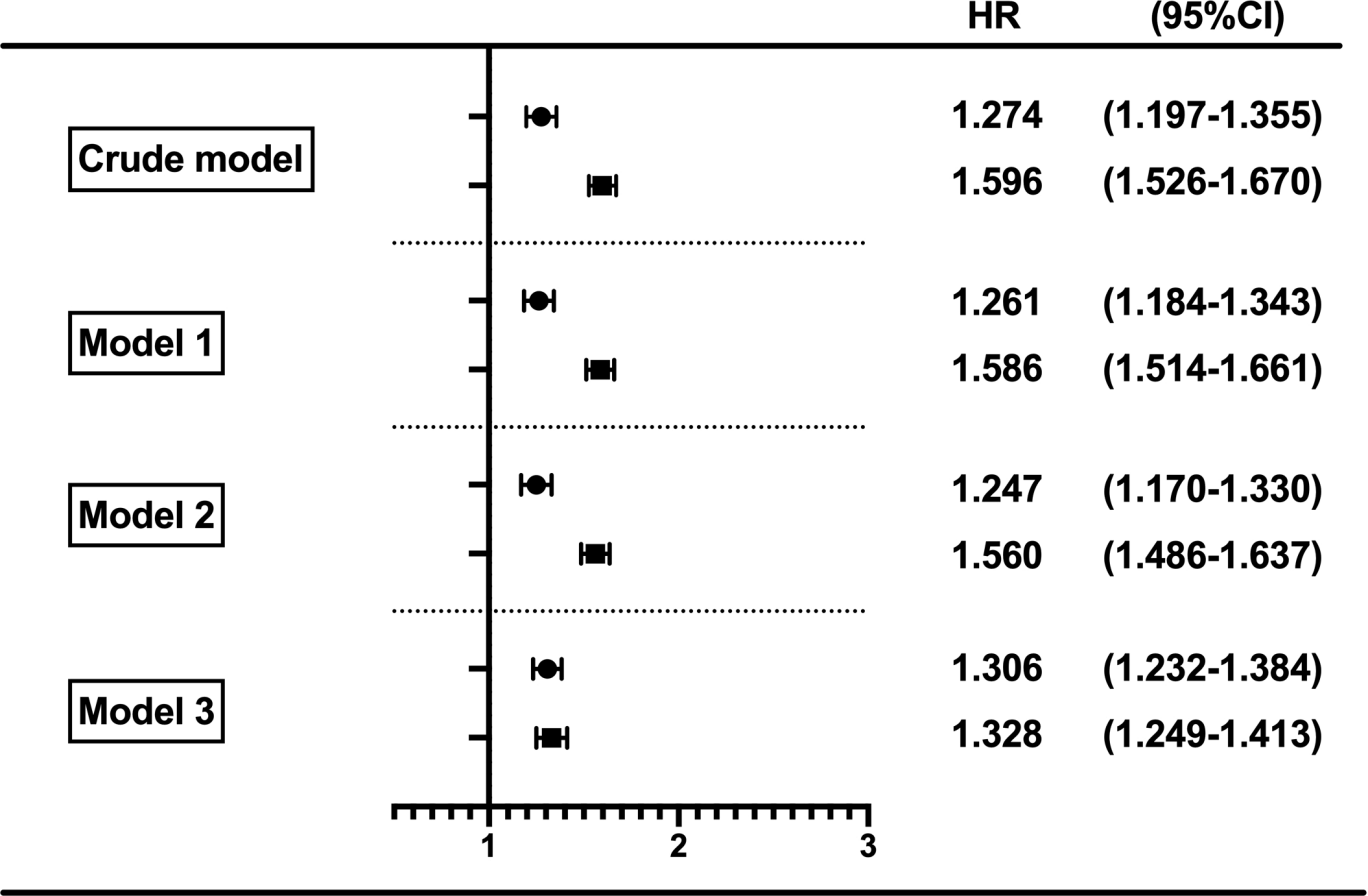
Forest plot of HRs for incident diabetes per SD difference in HGI and HbA1c. Circles represent HGI, and squares represent HbA1c in different Cox regression models. Model 1 was adjusted for age and sex. Model 2 was adjusted for the variables in model 1 plus body mass index, systolic blood pressure, diastolic blood pressure, hypertension, antihypertensive medication, total cholesterol, triglycerides, and low-density lipoprotein cholesterol. Model 3 was adjusted for all variables in model 2 plus the baseline fasting plasma glucose and 2-h plasma glucose at the OGTT. CI, confidence interval; HbA1c, hemoglobin A1c; HGI, hemoglobin glycation index; HR, hazard ratios; OGTT, oral glucose tolerance test.

Moreover, when considering the HGI as a continuous variable, every 1-SD increase resulted in a 30.6% increase in the risk of diabetes. In contrast, when the HbA1c level was used, every 1-SD increase resulted in a 32.8% increase in the risk of diabetes (**Figure 4**), and there were no differences between HGI and HbA1c with an overlap in the 95% CIs in the adjusted HRs.

## Discussion

In this prospective population-based cohort study, we found that regardless of baseline glycemic status, participants with higher HGI values were at a greater risk of incident diabetes during the 3-year follow-up period. This association remained significant even after adjustment for potential confounders of diabetes. Our study contributes new knowledge about the relative importance of inter-individual biological variation of HbA1c on predicting the risk of incident diabetes.

Pervious investigators have reported that two major factors determined HbA1c levels: the mean blood glucose level over an 8- to 12-week period and biological variation in HbA1c, as assessed by the HGI (3, 6, 7). The HGI quantifies the persistent individual discrepancy between HbA1c values and similar blood glucose levels, which is a good indicator to investigate the clinical implications of biological variation in HbA1c. To date, several reports have shown that a high HGI is significantly associated with an increased risk of developing both diabetic microvascular and macrovascular complications (12, 13, 19, 20).

However, few studies have focused on the implications of the HGI for the predictive value of incident diabetes. In this study, we used baseline HbA1c and FPG levels to show that a high HGI at baseline was related to a high risk of incident diabetes. At the 3-year follow-up, participants in the high and moderate HGI groups had a 1.903- and 1.366-fold greater risk of diabetes, respectively, than those in the low HGI group. Thus, individuals with a high HGI (i.e., high propensity for glycation) have a greater susceptibility to future diabetes at similar baseline glucose levels than those with a low HGI. Notably, these findings have ruled out the confounding influence of glycemia, which could underestimate or mask the true effect of the HGI on diabetes risk. To the best of our knowledge, this is the first study to explore the impact of high HGI on the progression of hyperglycemia.

When evaluating HGI as a continuous variable, every 1-SD increase was associated with a 30.6% increase in the risk of incident diabetes; using HbA1c instead of the HGI as a predictive variable to compare the effect on diabetes occurrence corroborated this result. After adjustment for identical potential confounders (especially blood glucose levels), every 1-SD increase in HbA1c resulted in a significant 32.8% increase in the risk of future diabetes, which was consistent with the result of the HGI assessment. The adjusted HRs of the HGI and HbA1c values were approximately the same, which supported the concept that the predictive value of the HGI reflected the non-glycemic predictive value of HbA1c. That is, except for the predictive value of glycemic measures, an individual’s propensity for glycation also has distinct effects on the development and progression of diabetes. The underlying mechanism linking a high HGI and glycemic disorders has not yet been fully elucidated. A high HGI, representing an increased degree of intracellular non-enzymatic glycosylation, is correlated with inflammation and the generation of AGEs (10, 11), both of which contribute to insulin resistance and impaired beta-cell function, thus causing elevated blood glucose levels (21–23). However, hyperglycemia could also cause chronic inflammation and increased generation of AGEs (24–26), thereby establishing a vicious cycle. In brief, a high HGI identified individuals with increased susceptibility to diabetes and associated complications at similar blood glucose concentrations.

Previous studies have reported that individuals with a high HGI exhibit similar clinical traits, including older age, a higher BMI, dyslipidemia, and postprandial glycemic excursion (9, 15, 27–29). However, the observations from different studies are not completely concordant. These discrepancies might be attributed to the different geographical populations with different study inclusion criteria and different methods of calculating HGI. Accordingly, further studies are needed to unify and standardize the calculating method of HGI and confirm which clinical features are most highly associated with higher HGI.

HGI is a marker with biological variation in HbA1c or an individual’s propensity for glycation, combined with HbA1c, which might provide a better assessment of a patient’s risk for glycemic progression. This prospective cohort study showed that high HGI was associated with an increased risk for diabetes. Therefore, our findings will facilitate work to understand the linkage between an individual’s high propensity for glycation and predisposition to hyperglycemia.

The potential limitations of our study are as follows: First, the diagnosis of diabetes was based on a single measurement of glucose (FPG or 2hPG during OGTT) or HbA1c, which may lead to misdiagnosis or misclassification for some patients without classic symptoms of hyperglycemia. Second, the study participants originated from a single center in the REACTION study; thus, the results may not be directly generalizable, and further studies are required to confirm our findings.

In summary, our study findings suggest that inter-individual differences in the propensity for glycation assessed by the HGI are associated with the risk of incident diabetes. Hence, more attention should be given to the development and progression of glycemic abnormalities in participants with a high HGI. In addition, the application of HbA1c to assess average glycemia in considering individual HGI levels may help to individualize preventive and therapeutic decision-making. Also, in constructing a population-based prediction model, the HGI measurement may be considered an additional parameter to improve the risk stratification of patients with diabetes. Consequently, future work should focus on investigating the underlying mechanism of how the HGI involves the pathogenesis of diabetes and how to reduce the risk of developing diabetes in a population with high HGIs.

## Data availability statement

The datasets presented in this article are not readily available because of individual privacy reasons, but are available after anonymization from the corresponding author on reasonable request. Requests to access the datasets should be directed to jingtaodou@163.com.

## Ethics statement

The studies involving human participants were reviewed and approved by the Medical Ethics Committee of Shanghai Jiaotong University (No. 2011-14). The patients/participants provided their written informed consent to participate in this study.

## Author contributions

LL and JD contributed to this study’s concept, design, implementation, and rationale. LL performed the statistical analysis with advice from YH. LL drafted and revised the manuscript. JD supervised the study, and revised the paper. All authors contributed to the acquisition, analysis, and interpretation of the data and the review and edits of the drafts. JD is responsible for the integrity of the work as a whole. All authors contributed to the article and approved the submitted version.
