# Supplementary material for: High hemoglobin glycation index is associated with increased risk of diabetes: A population-based cohort study in China
Source: Front Endocrinol (Lausanne). 2023 Feb 27;14:1081520. doi: 10.3389/fendo.2023.1081520 (PMC9999023; doi:10.3389/fendo.2023.1081520)
Supplement: Supplementary file 1 [file Table_1.docx]

Table S1 Baseline Characteristics of normoglycemia participants in the three HGI groups

| variables | total | Low HGI | Moderate HGI | High HGI | Statistical value | P value |
| --- | --- | --- | --- | --- | --- | --- |
| Participants, n | 4106 | 1236 | 1509 | 1361 |  |  |
| Mean HGI | 0.0185 (-0.2055, 0.2485)) | -0.3233 (-0.4670, -0.2335) | 0.0070 (-0.0715, 0.0835) | 0.3495 (0.2495, 0.4907) |  |  |
| Age (years) | 55.6±7.3 | 54.7±6.9 | 55.8±7.5 | 56.3±7.2 | 18.174 | ＜0.001 |
| Female, n(%) | 2812 (68.5) | 791 (64.0) | 1058 (70.1) | 963 (70.8) | 16.643 | ＜0.001 |
| BMI (kg/m^2^) | 25.0±3.3 | 24.7±3.2 | 25.0±3.2 | 25.2±3.4 | 7.870 | ＜0.001 |
| Waist circumference (cm) | 81.6±8.8 | 81.9±8.8 | 81.5±8.6 | 81.6±9.0 | 0.742 | 0.476 |
| SBP (mmHg) | 127.6±15.4 | 127.2±15.6 | 127.5±15.5 | 128.2±15.1 | 1.359 | 0.257 |
| DBP (mmHg) | 74.6±9.4 | 75.7±9.4 | 74.4±9.3 | 73.9±9.3 | 12.492 | ＜0.001 |
| Family history of diabetes, n(%) | 907 (22.1) | 254 (20.6) | 337 (22.3) | 316 (23.2) | 4.377 | 0.357 |
| History of hypertension, n(%) | 913 (22.2) | 255 (20.6) | 342 (22.7) | 316 (23.2) | 2.760 | 0.252 |
| TC (mmol/L) | 5.16±0.94 | 5.00±0.89 | 5.20±0.92 | 5.27±0.98 | 33.828 | ＜0.001 |
| TG (mmol/L) | 1.18 (0.85, 1.66) | 1.13 (0.82, 1.59) | 1.20 (0.85, 1.70) | 1.21 (0.89, 1.73) | 14.072 | 0.001 |
| HDL-C (mmol/L) | 1.44 (1.22, 1.70) | 1.43 (1.21, 1.70) | 1.45 (1.22, 1.69) | 1.44 (1.21, 1.74) | 1.084 | 0.582 |
| LDL-C (mmol/L) | 3.14±0.77 | 3.02±0.73 | 3.17±0.77 | 3.23±0.81 | 26.225 | ＜0.001 |
| HbA1c (%) | 5.7±0.4 | 5.2±0.3 | 5.6±0.2 | 6.0±0.3 | 2058.508 | ＜0.001 |
| FPG (mg/dl) | 92±5 | 92±5 | 92±6 | 91±6 | 15.480 | ＜0.001 |
| 2hPG (mg/dl) | 108±19 | 107±19 | 108±19 | 108±19 | 1.345 | 0.261 |

Data are expressed as mean ± standard deviation for normally distributed variables, median (P_25_, P_75_) for non-normally distributed variables, and number (%) for categorical variables.

BMI, body mass index; DBP, diastolic blood pressure; FPG, fasting plasma glucose; HbA1c, glycated hemoglobin A1c; HDL-C, high-density lipoprotein cholesterol; HGI, hemoglobin glycation index; LDL-C, low-density lipoprotein cholesterol; SBP, systolic blood pressure; TC, total cholesterol; TG, triglycerides; 2hPG: 2-h plasma glucose

Table S2 Baseline Characteristics of pre-diabetes participants in the three HGI groups

| variables | total | Low HGI | Moderate HGI | High HGI | Statistical value | P value |
| --- | --- | --- | --- | --- | --- | --- |
| Participants, n | 3239 | 1224 | 1033 | 982 |  |  |
| Mean HGI | -0.0250 (-0.2775, 0.2270) | -0.3615 (-0.5185, -0.2422) | 0.0090 (-0.0650, 0.0840) | 0.3725 (0.2630, 0.5430) |  |  |
| Age (years) | 57.7±7.8 | 56.8±7.7 | 57.7±7.8 | 58.7±7.8 | 17.620 | ＜0.001 |
| Female, n(%) | 2073 (64.0) | 700 (57.3) | 715 (69.1) | 658 (66.9) | 38.862 | ＜0.001 |
| BMI (kg/m^2^) | 26.2±3.3 | 25.7±3.2 | 26.3±3.4 | 26.7±3.4 | 22.204 | ＜0.001 |
| Waist circumference (cm) | 84.7±8.4 | 84.3±8.3 | 84.7±8.3 | 85.4±8.5 | 4.695 | 0.009 |
| SBP (mmHg) | 133.7±16.3 | 133.8±16.2 | 132.8±16.1 | 134.4±16.6 | 2.441 | 0.087 |
| DBP (mmHg) | 76.7±9.6 | 77.8±9.6 | 76.3±9.4 | 75.7±9.6 | 14.712 | ＜0.001 |
| Family history of diabetes, n(%) | 865 (26.7) | 341 (27.9) | 288 (27.8) | 236 (24.0) | 5.337 | 0.254 |
| History of hypertension, n(%) | 1058 (32.7) | 398 (32.6) | 305 (29.5) | 355 (36.1) | 10.127 | 0.006 |
| TC (mmol/L) | 5.32±1.02 | 5.16±0.96 | 5.37±1.11 | 5.45±0.98 | 24.460 | ＜0.001 |
| TG (mmol/L) | 1.41 (1.00, 1.99) | 1.32 (0.96, 1.88) | 1.44 (0.98, 2.01) | 1.48 (1.09, 2.07) | 26.012 | ＜0.001 |
| HDL-C (mmol/L) | 1.37 (1.15, 1.61) | 1.37 (1.15, 1.62) | 1.38 (1.17, 1.62) | 1.35 (1.14, 1.60) | 2.298 | 0.317 |
| LDL-C (mmol/L) | 3.27±0.82 | 3.15±0.81 | 3.30±0.82 | 3.39±0.81 | 24.037 | ＜0.001 |
| HbA1c (%) | 5.9±0.5 | 5.6±0.3 | 5.9±0.2 | 6.4±0.4 | 1300.646 | ＜0.001 |
| FPG (mg/dl) | 104±9 | 106±8 | 103±8 | 102±9 | 63.761 | ＜0.001 |
| 2hPG (mg/dl) | 143±29 | 137±29 | 143±28 | 149±28 | 45.968 | ＜0.001 |

Data are expressed as mean ± standard deviation for normally distributed variables, median (P_25_, P_75_) for non-normally distributed variables, and number (%) for categorical variables.

BMI, body mass index; DBP, diastolic blood pressure; FPG, fasting plasma glucose; HbA1c, glycated hemoglobin A1c; HDL-C, high-density lipoprotein cholesterol; HGI, hemoglobin glycation index; LDL-C, low-density lipoprotein cholesterol; SBP, systolic blood pressure; TC, total cholesterol; TG, triglycerides; 2hPG: 2-h plasma glucose
